# Hydrogeochemical data on groundwater quality with special emphasis on fluoride enrichment in Munneru river basin (MRB), Telangana State, South India

**DOI:** 10.1016/j.dib.2018.01.059

**Published:** 2018-01-31

**Authors:** Adimalla Narsimha, S. Venkatayogi, S. Geeta

**Affiliations:** a School of Environmental Science and Engineering, Chang'an University, No. 126 Yanta Road, Xi’an 710054, China; b Key Laboratory of Subsurface Hydrology and Ecological Effects in Arid Region of the Ministry of Education, Chang'an University, No. 126 Yanta Road, Xi’an 710054, Shaanxi, China; c Department of Applied Geochemistry, University College of Science, Osmania University, Hyderabad 500007, India; d Department of Chemistry, MVSR Engineering College, Hyderabad 501510, India

## Abstract

Fluorosis is one of the most prevailing groundwater related disease in developing countries like India and China. In India, 20 out of 29 states have some extent of groundwater fluoride contamination. In especially, Telangana State all (10 out of 10) districts are fluoride affected (Adimalla and Venkatayogi, 2017) [2]. However, this article describes about fluoride contamination and correlation between fluoride and other hydrochemical parameters, in the Munneru river basin (MRB) groundwater, Telangana State, South India. The fluoride concentration in groundwater of Munneru river basin ranged from 0.3 to 8.0 mg/L, with a mean of 1.607 mg/L. About 35% of the groundwater samples have fluoride concentration above > 1.5 mg/L which are unsuitable for drinking purposes. However, 53% of groundwater locations are within the acceptable limits (0.5–1.5 mg/L) and these are very suitable for drinking purposes and remaining 22% of collected groundwater samples were having less than the required limit of 0.5 mg/L.

**Specifications Table**

**Table:** 

Subject area	*Environmental Sciences*
More specific subject area	*Hydro-geochemistry*
Type of data	*Table, figure*
How data was acquired	*The fluoride concentration in groundwater was determined electrochemically, using a fluoride ion-selective electrode (ISE) with an Orion 4 star meter benchtop pH/ISE meter.*
Data format	*Analyzed*
Experimental factors	*All groundwater samples were collected in pre-washed polyethylene narrow-mouth bottles polyethylene bottles and stored in a dark place at room temperature until the fluoride and other physiochemical analysis.*
Experimental features	*Determine the content levels of fluoride and other Physiochemical parameters using standard procedure.*
*Data source location*	*MRB, longitude: 79.82798633 to 79.93446207; latitudes: 17.87285336 to 17.9557804, Telangana State, South India.*
Data accessibility	*Data is with this article*

**Value of the data**

• The data which were presented here can be a pave path to design a sustainable planning and management of the groundwater resource to protect and supply potable water to dependent population.
• In most of the arid and semi-arid regions, groundwater is the upper most source of water supply. Therefore, water quality is the most important in such regions and it brings health problems. Hence, continuous monitoring of the quality of water is very essential.
• Identification of groundwater vulnerability zones is the primary and taking necessary precautions is foremost vital, in order to protect future generations.
• This data will be useful to develop effective strategies for improving rural drinking water supply and provide scientific evidence for decision and management of the groundwater.

## 1 Data

**Fig. 1:**
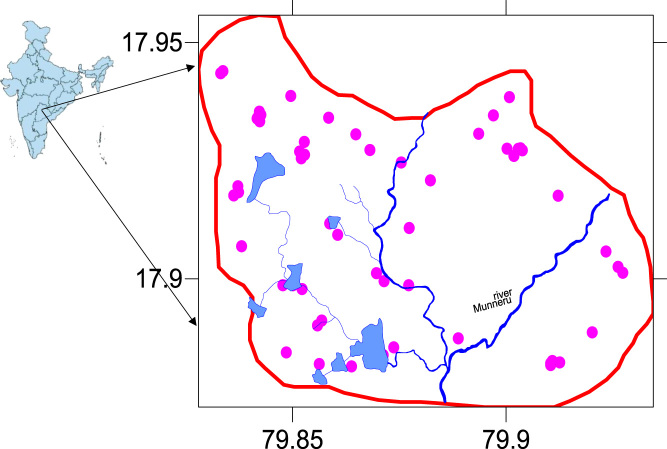
Location map of the groundwater samples from the Munneru river basin (MRB), Telangana State, South India.

**Fig. 2:**
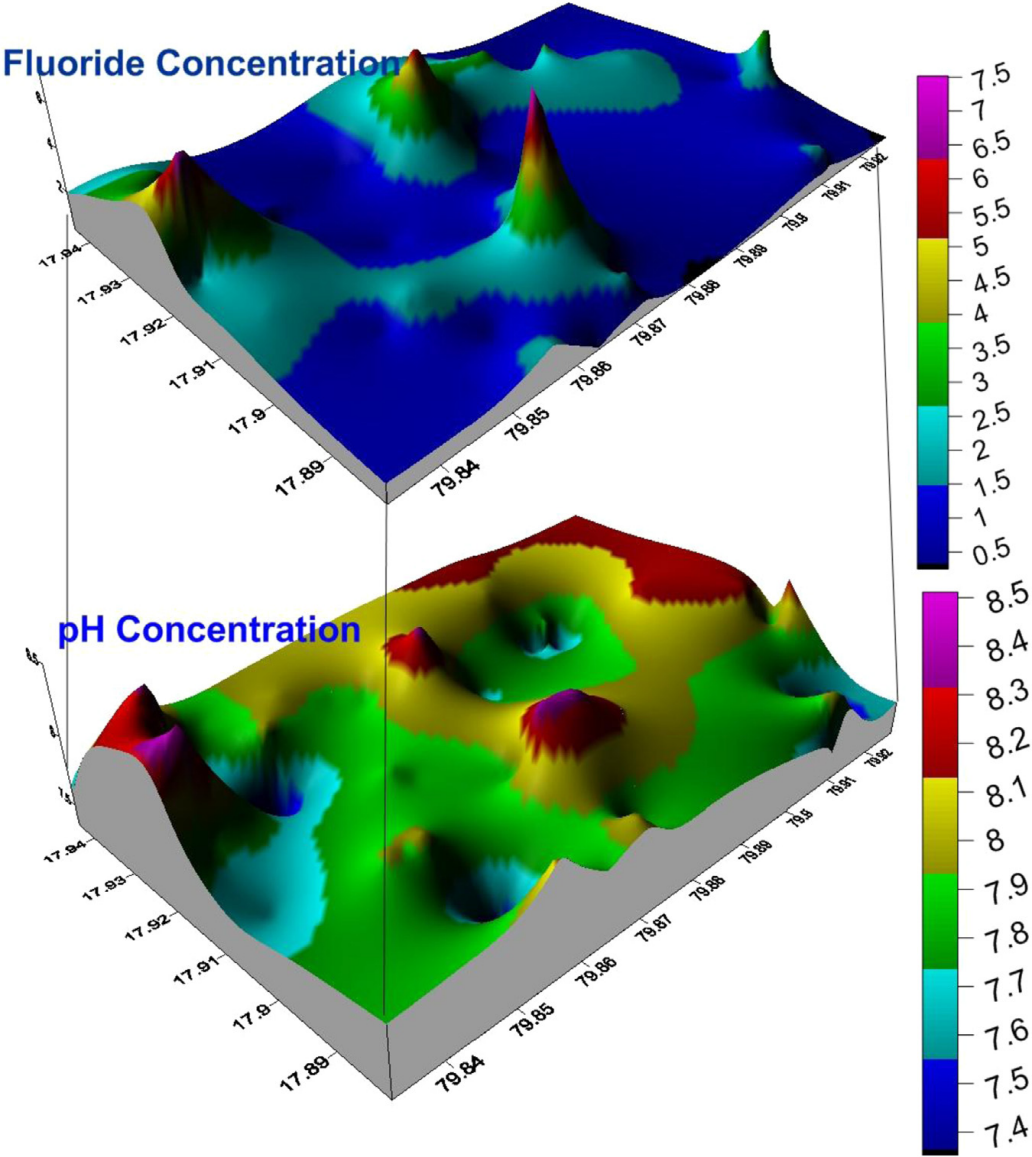
Three-dimensional distribution and correlation with pH and Fluoride concentration in Munneru river Basin (MRB), Telangana State, South India.

**Fig. 3:**
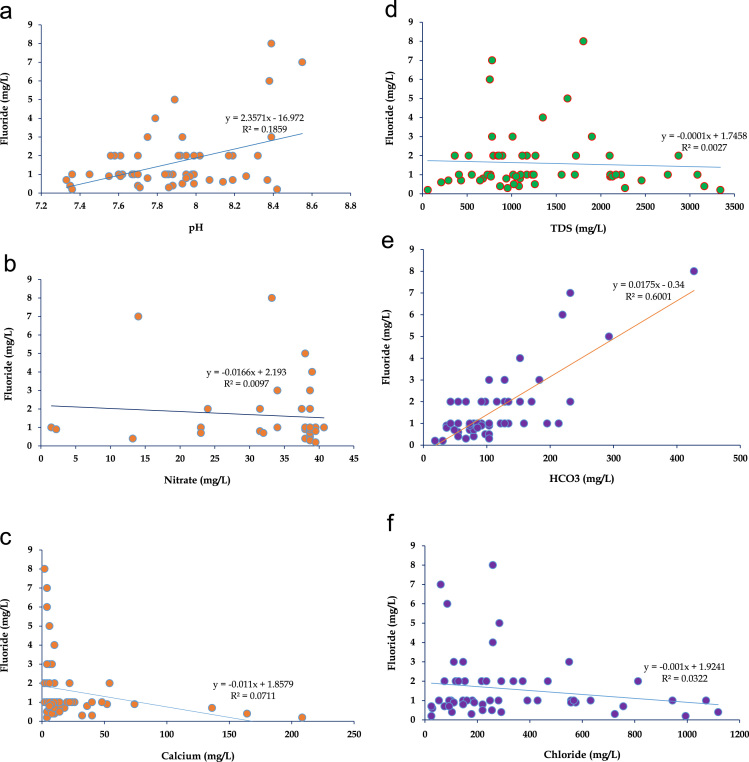
Bivariate scatter plots: (a) F^−^ versus pH, (b) F^−^ versus NO_3_^−^_,_ (c) F^−^ versus Ca^2+^ (d) F^−^ versus TDS (e) F^−^ versus HCO_3_^−^ and (f) F^−^ versus Cl^−^.

The MRB is one of the tributes of Krishna River, which is under arid and semi-arid condition and also MRB occupied by the Granitic rocks of Archaean age. The groundwater samples and its locations were shown in Fig. 1. The most of the MRB surrounding villagers depend on groundwater for their drinking and other house hold applications and to know the groundwater quality is the foremost vital and especially fluoride concentration, because it effects human health. The fluoride contents found in the groundwater samples range between 0.3 and 8.0 mg/L with a mean of 1.607 mg/L (Table 1, Table 2). The high fluoride content in the groundwater of MRB, which exceeds the maximum limit for drinking waters (1.5 mg/L) and east and south part of the MRB is shown elevated fluoride levels (Fig. 2). The fluoride ion (F^−^) is the most common form in which fluorine occurs in the environment, although its behavior in groundwater is strongly dependent on the pH, since it has a lower solubility at pH 6.0–6.5 [1], [2], [3], [4], [5], [6], [7], [8], [9], [10], [11], [12]. Binary diagrams of fluoride versus pH, nitrate, calcium, total dissolved solids (TDS), bicarbonate and chloride were performed to identify the groundwater influence factors, positive or negative correlations (Fig. 3).

**Table 1:** Results of the chemical analysis of groundwater samples collected from Munneru river basin (MRB), Telangana State, South India.

**Sample ID**	**pH**	**EC**	**TDS**	**TH**	**Ca**^**2+**^	**Mg**^**2+**^	**Na**^**+**^	**K**^**+**^	**Cl**^−^	**HCO₃**^−^	**NO**^−^	**SO**_**4**_^**2**^^−^	**F**^−^
		**µS/cm**	**mg/L**										
*MRBT-1	7.7	1400	896	125	4	28	52	2	152.6	98	39	30	2
MRBT-2	7.61	2380	362	195	10	41	79	3	337.2	98	39	34	2
MRBT-3	8.42	92.3	59	20	4	2.4	8	1	24.8	18	8	14	0.2
MRBT-4	7.85	1190	762	125	4	28	52	4	99.4	128	38	23	1
MRBT-5	7.99	1970	1260	195	4	45	68	5	255.6	98	35	56	0.5
MRBT-6	7.88	1360	870	190	10	40	26	3	102.9	79	39	53	0.4
MRBT-7	7.88	1830	1171	165	2	39	68	4	181.1	195	39	41	1
MRBT-8	8.38	1180	755	40	4	7.3	74	3	85.2	220	34	31	6
MRBT-9	7.93	1220	780	70	6	13	55	3	110.1	128	38	19	3
MRBT-10	8.07	67 5	432	100	18	13	30	7	74.6	79	33	8	0.7
MRBT-11	8.14	328	209	50	12	4.8	17	4	28.4	55	24	0	0.6
MRBT-12	7.62	2440	1561	315	26.1	61	56	4	429.5	92	35	8	1
MRBT-13	7.58	4490	2873	555	54.1	102	100	5	812.9	67	39	37	2
MRBT-14	7.95	1060	678	110	14	17	38	13	145.5	79	32	15	0.8
MRBT-15	7.45	865	553	55	8	8.5	44	6	92.3	73	2	26	1
MRBT-16	7.68	1140	729	80	4	17	35	35	53.3	159	23	31	1
MRBT-17	7.75	2970	1900	350	8	80	75	4	550.2	104	34	34	3
MRBT-18	8.19	810	518	80	6	16	39	3	74.5	92	24	15	2
MRBT-19	8.19	453	290	55	6	10	18	4	24.8	73	23	35	0.7
MRBT-20	7.79	2110	1350	135	10	27	79	3	259.1	153	39	45	4
MRBT-21	8.26	1200	768	125	6	27	32	4	110.1	104	38	19	0.9
MRBT-22	7.95	1590	1018	205	12	43	32	4	145.5	79	39	31	1
MRBT-23	7.55	3290	2106	365	74.1	45	52	6	575.1	37	39	49	0.9
MRBT-24	7.95	1240	794	110	10	21	49	4	117.2	55	39	15	2
MRBT-25	7.75	1710	1094	215	36.1	30	41	5	220	37	39	37	0.8
MRBT-26	7.35	4940	3162	705	164.3	72	76	25	1118.2	55	38	94	0.4
MRBT-27	7.7	1690	1082	215	8	47	36	5	291.1	79	13	26	0.4
MRBT-28	7.98	639	409	110	6	23	22	5	53.2	73	34	4	1
MRBT-29	8.37	1010	646	105	10	19	33	6	92.3	79	32	26	0.7
MRBT-30	8.39	2820	1805	115	2	27	125	5	259.1	427	33	71	8
MRBT-31	7.86	3290	2106	310	6	72	99	7	557.3	214	39	41	1
MRBT-32	7.85	3480	2227	450	14	101	79	9	631.9	122	41	45	1
MRBT-33	7.93	1600	1024	200	14	40	50	5	220.1	104	39	34	0.5
MRBT-34	7.71	3550	2272	375	40.1	67	86	5	724.2	104	39	56	0.3
MRBT-35	8.02	1830	1171	110	2	26	84	4	216.5	232	38	34	2
MRBT-36	7.99	1920	1229	100	6	21	86	5	248.5	134	38	19	1
MRBT-37	7.86	1490	954	220	32.1	34	27	5	177.5	67	39	34	0.3
MRBT-38	7.33	3840	2458	560	136.2	54	56	7	756.2	49	39	75	0.7
MRBT-39	7.6	1950	1248	225	20	43	56	5	280.5	85	39	37	1
MRBT-40	8.17	1320	845	135	10	27	56	5	74.6	128	39	26	2
MRBT-41	7.84	1720	1101	170	10	35	60	5	159.8	128	40	53	1
MRBT-42	7.99	1750	1120	155	6	34	65	2	234.3	134	39	37	2
MRBT-43	7.92	3280	2099	170	4	39	119	4	468.6	153	39	68	2
MRBT-44	7.97	1640	1050	120	8	24	66	2	188.2	92	2	37	0.9
MRBT-45	7.56	1980	1267	210	22.1	38	62	3	291.1	43	39	23	2
MRBT-46	7.91	2690	1722	100	6	21	112	2	372.8	171	32	64	2
MRBT-47	7.89	2540	1626	155	6	34	104	2	284	293	38	45	5
MRBT-48	8.55	1220	781	50	4	10	83	1	60.4	232	14	23	7
MRBT-49	7.36	2670	1709	310	24.1	61	63	3	390.5	43	40	56	1
MRBT-50	7.36	5220	3341	815	208.4	72	63	2	994	31	40	56	0.2
MRBT-51	7.61	3330	2131	450	52.1	78	60	2	557.4	73	39	30	0.9
MRBT-52	7.95	1470	941	120	6	26	61	1	145.6	85	40	30	0.8
MRBT-53	8.39	1580	1011	125	4	28	73	2	145.6	183	39	15	3
MRBT-54	7.67	4820	3085	555	40	111	97	2	1072.1	104	39	53	1
MRBT-55	8.32	1340	858	165	6	36	45	2	127.8	116	39	41	2
MRBT-56	7.68	4300	2752	400	48.1	68	105	3	944.3	73	39	30	1
MRBT-57	7.7	3390	2170	215	22.1	39	100	3	568	55	39	101	1

^⁎^MRBT: Munneru river basin groundwater, Telangana State

**Table 2:** Statistical summary of the chemical composition of groundwater from Munneru river basin (MRB), Telangana State, South India.

**Water quality parameters**		**Minimum**	**Maximum**	**Mean**	**Median**	**Standard deviation**	**Coefficient of variation**	**Acceptable limit**	**Permissible limit**	**Undesirable effect**
**pH**		7.33	8.55	7.882	7.88	0.292	0.037	6.5–8-5	No relaxation	Taste
**EC**	µS/cm	92.3	5220	2118.345	1735	1209.651	0.571	–	1500	Gastrointestinal irritation
**TDS**	mg/L	59	3341	1319.123	1094	787.263	0.597	500	2000	Gastrointestinal irritation
**TH**		20	815	214.912	165	169.475	0.789	200	600	–
**Ca**^**2+**^		2	208.4	22.807	10	38.660	1.695	75	200	Scale formation
**Mg**^**2+**^		2.4	111	38.474	34	24.903	0.647	30	100	–
**Na**^**+**^		8	125	61.895	60	27.232	0.440	–	200	High blood pressure
**K**^**+**^		1	35	4.965	4	5.318	1.071	–	12	Bitter taste
**Cl**^**−**^		24.8	1118.2	311.712	220	281.365	0.903	250	1000	Salty taste
**HCO**_**3**_^**−**^		18.3	427	111.512	91.5	70.779	0.635			–
**NO**^**−**^		1.5	40.7	33.970	38.7	9.327	0.275	45	No relaxation	methemoglobinemia
**SO**_**4**_^**2**^^**−**^		0	101.2	36.556	33.5	20.395	0.558	200	400	Laxative effect
**F**^**−**^		0.2	8	1.607	1	1.595	0.993	1	1.5	Fluorosis

## 2 Experimental design, materials, and methods

### 2.1 The study area description

**Fig. 4:**
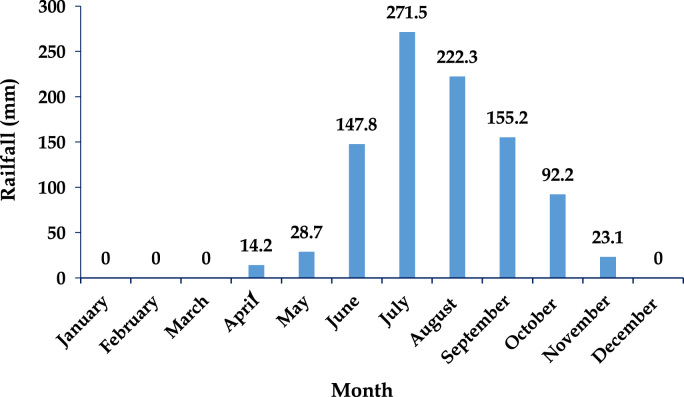
Mean monthly rainfall distribution in MRB, Warangal rural district, Telangana State, South India.

Munneru river basin (MRB) stretches geo-graphically from 79.82798633 to 79.93446207 E longitude and 17.87285336 to 17.9557804 N latitude, positioned in the Warangal rural district, in the eastern part of Telangana (Fig. 1) and the mean monthly rainfall distribution is in shown in Fig. 4.

### 2.2 Sample collection and analysis procedure

Fifty seven groundwater samples were collected in pre-cleaned and sterilized polyethylene bottles of 1 L capacity with necessary precautions [13]. The groundwater samples were analyzed for various hydro chemical parameters such as pH, electrical conductivity (EC), total hardness (TH) as CaCO3, calcium (Ca^2+^), magnesium (Mg^2+^), sodium (Na^+^), potassium (K^+^), chloride (Cl^−^), sulphate (SO_4_^2^^−^), nitrate (NO_3_^−^) and fluoride (F^−^) [13] and detailed analysis procedure is depicted in Table 3. The fluoride concentration in water was determined electrochemically, using fluoride in selective electrode [13]. This method is applicable to the measurement of fluoride in drinking water in the concentration range of 0.01–1000 mg/L. The electrode used was an Orion fluoride electrode, coupled to an Orion electrometer. Standards fluoride solutions (0.1–10 mg/L) were prepared from a stock solution (100 mg/L) of sodium fluoride. As per experimental requirement, 1 ml of Total Ionic strength Adjusting Buffer Grade III (TISAB III) was added in 10 ml of sample. The ion meter was calibrated for a slop of − 59.2 ± 2 [12]. The composition of TISAB solution was as 385.4 g ammonium acetate, 17.3 g of cyclohexylene diamine tetraacetic acid (CDTA) and 234 ml of concentrate hydrochloric acid per liter.

**Table 3:** Instrumental, titrimetric and calculation methods used for hydrochemical analysis of groundwater samples from *Munneru river basin (MRB)*, Telangana State, South India.

*Parameters*	*Characteristics*	*Analytical method*	*Unit*	*Reference*
*General*	pH	pH/EC/TDS meter	–	APHA 1995
	Electrical Conductivity	pH/EC/TDS meter	µS/cm	APHA 1995
	Total dissolved Solids (TDS)	ECX(0.55 to 0.75)	mg/L	Hem 1991
	Total hardness (as CaCO_3_)	EDTA titrimetric	mg/L	APHA 1995
*Major Cations*	Calcium (as Ca^2+^)	EDTA titrimetric	mg/L	APHA 1995
	Magnesium (as Mg^2+^)	Calculation (TH-Ca^2+^)	mg/L	APHA 1995
	Sodium (as Na^+^)	Flame photometric	mg/L	APHA 1995
	Potassium (as K^+^)	Flame photometric	mg/L	APHA 1995
*Major anions*	Bicarbonates (HCO_3_^-^)	Titrimetric	mg/L	APHA 1995
	Chloride (Cl^−^)	AgNO_3_ titrimetric	mg/L	APHA 1995
	Fluoride (F^−^)	ISE (Ion selective electrode)	mg/L	APHA 1995
	Nitrate (NO_3_^−^)	UV visible spectrophotometer	mg/L	APHA 1995
	Sulphates (SO_4_^2^^−^)	UV visible spectrophotometer	mg/L	APHA 1995
