# Supplementary material for: Hydrogeochemical data on groundwater quality with special emphasis on fluoride enrichment in Munneru river basin (MRB), Telangana State, South India
Source: Data Brief. 2018 Jan 31;17:339–46. doi: 10.1016/j.dib.2018.01.059 (PMC5988497; doi:10.1016/j.dib.2018.01.059)
Supplement: Supplementary file 1 — Transparency document [file mmc1.doc]

No conflict interest
